# Comparative study of percutaneous nephrolithotomy performed in the traditional prone position and in three different supine positions

**DOI:** 10.1590/S1677-5538.IBJU.2018.0191

**Published:** 2019

**Authors:** Petronio Augusto de Souza Melo, Fabio Carvalho Vicentini, Rodrigo Perrella, Claudio Bovolenta Murta, Joaquim Francisco de Almeida Claro

**Affiliations:** 1 Divisão de Urologia do Centro de Saúde Masculina do Hospital Brigadeiro, São Paulo, SP, Brasil

**Keywords:** Nephrolithotomy, Percutaneous, Prone Position, Kidney Calculi

## Abstract

**Purpose:**

To compare the outcomes of percutaneous nephrolithotomy (PCNL) performed in the prone position (PRON) and in three variations of the supine position.

**Materials and Methods:**

We performed a retrospective analysis of patients that underwent PCNL at our institution from June 2011 to October 2016 in PRON and in three variations of the supine position: complete supine (COMPSUP), original Valdivia (VALD), and Galdakao - modified Valdivia (GALD). All patients had a complete pre - operative evaluation, including computed tomography (CT). Success was defined as the absence of residual fragments larger than 4 mm on the first post - operative day CT.

**Results:**

We analyzed 393 PCNLs: 100 in COMPSUP, 94 in VALD, 100 in GALD, and 99 in PRON. The overall success rate was 50.9% and was similar among groups (p = 0.428). There were no differences between groups in the number of punctures, stone - free rate, frequency of blood transfusions, drop in hemoglobin level, length of hospital stay, and severe complications (Clavien ≥ 3). COMPSUP had a significantly lower operative time than the other positions. COMPSUP had lower fluoroscopy time than VALD.

**Conclusion:**

Patient positioning in PCNL does not seem to impact the rates of success or severe complications. However, COMPSUP is associated with a shorter surgical time than the other positions.

## INTRODUCTION

Percutaneous nephrolithotomy (PCNL) has been performed since 1976 (1), and it is currently the gold - standard procedure for large renal calculi (2). Initially, percutaneous access to the kidney was only performed in the prone position, as described sixty years ago (3). The first description of supine positioning in PCNL was from Valdivia Uria et al. (4). Since then, both positions have been adopted for PCNL.

Modifications of the original supine position were developed to improve the surgical outcomes. Kumar et al. (5) reviewed five different supine positions (complete supine, Valdivia, Galdakao - modified Valdivia, Barts - modified Valdivia, and Barts flank - free modified supine positions) and presented the merits of each supine position. They concluded that there was no one superior supine position. Rather, the best position depended on the patient and their specific stone burden. Kumar also discussed the lack of high level evidence comparing the various positions used for supine PCNL.

No consensus exists on the best positioning in PCNL, nor is it clear which variation of the supine position is best. The aim of this study was to evaluate outcomes of PCNL in the prone position (PRON) and in three different variations of the supine position: complete supine position (COMPSUP), original Valdivia supine position (VALD), and Galdakao - modified supine Valdivia (GALD).

## MATERIALS AND METHODS

We performed a retrospective analysis of our prospectively collected database of patients who underwent PCNL between June 2011 and October 2016. Indications of surgery were renal stones ≥ 2 cm or symptomatic calculi < 2 cm in which first - line techniques (shock wave lithotripsy or uretero - renoscopy) failed.

Patients underwent PCNL in PRON or one of the supine position variations (COMPSUP, VALD, or GALD) commonly used at our institution. Positioning of was solely based on surgeon preference. The surgeons were trained in all positions.

During this study period, we performed 1.066 PCNL in four positions, including COMPSUP (n = 699), VALD (n = 111), GALD (n = 141), and PRON (n = 115). We chose 100 patients from each group distributed equally over the collection period. Patients presenting with associated ureteral calculi and incomplete data were excluded from the analysis.

All patients had a non - contrast - enhanced computed tomography (CT) prior to the surgery and on the first post - operative day (POD) to verify residual fragments (RF) and evaluate surgical complications.

Variables analyzed included the number of puncture tracts, operative time, fluoroscopy time, success rate, drop of hemoglobin level, need for blood transfusion, complications, length of hospital stay, and tubeless rate. All cases were graded by two urologists using Guy's Stone Score (GSS) (6) based on CT findings. In cases with different opinions, the final grade was determined by the most experienced urologist. Operative time was defined as the beginning of cystoscopy until last nephroscopy or insertion of a nephrostomy tube. We used the 4 mm threshold to define success based on a study by Raman et al. (7) demonstrating that second - look flexible nephroscopy is not cost advantageous for RF ≤ 4 mm. Stone - free was defined as the absence of any RF. Complications were classified according to the Clavien - Dindo grading system (8, 9).

### PCNL technique

**Figure 1:**
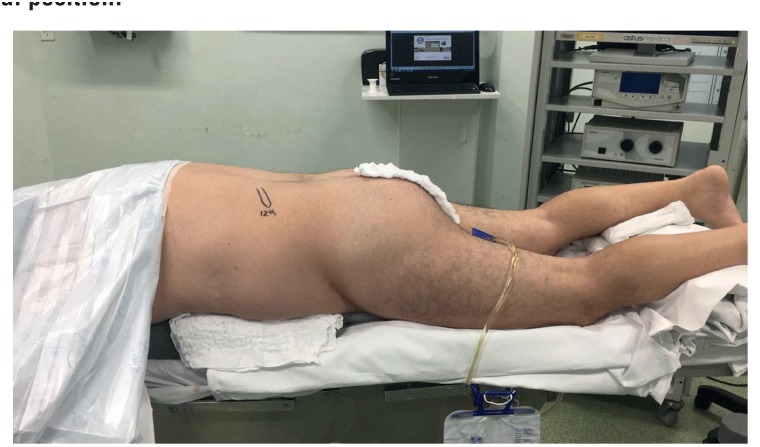
Ventral position.

The PRON (Figure-1) technique followed these classic steps: patients were placed in a lithotomy position and a ureteral catheter was inserted through a rigid cystoscope to perform a retrograde pyelogram. The ureteral catheter was fixed to a Foley catheter, and then the patient was repositioned to the prone position with pads under their shoulders (10).

**Figure 2:**
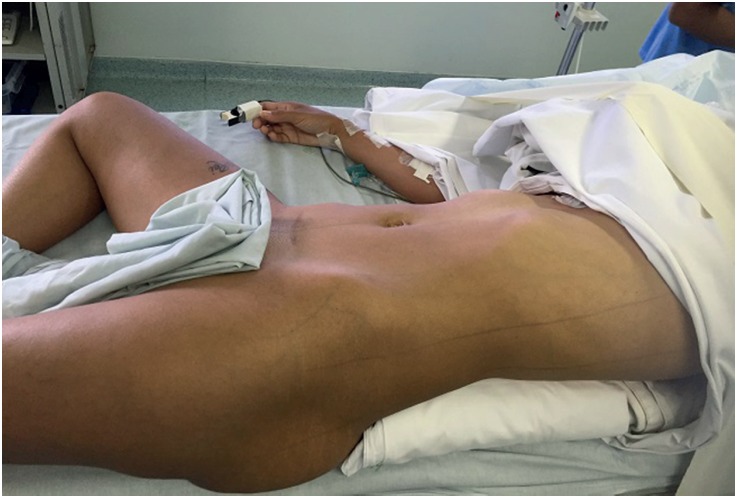
Valdivia position.

When we used VALD (Figure-2) (11), patients were initially in the lithotomy position, the ureteral catheter was inserted through a rigid cystoscope, then the stirrups were removed and the patient was left in supine decubitus with a 3 - liter saline bag below the ipsilateral flank.

**Figure 3:**
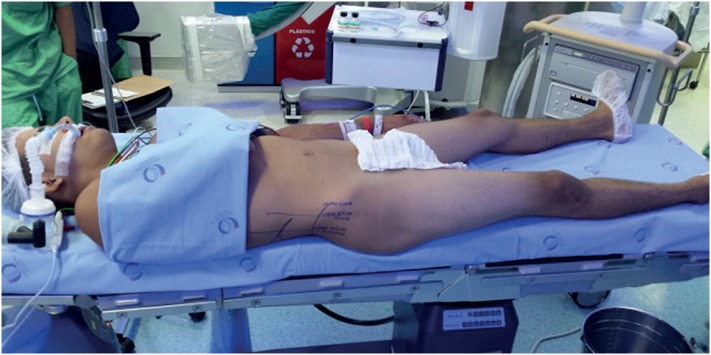
Complete supine position.

**Figure 4:**
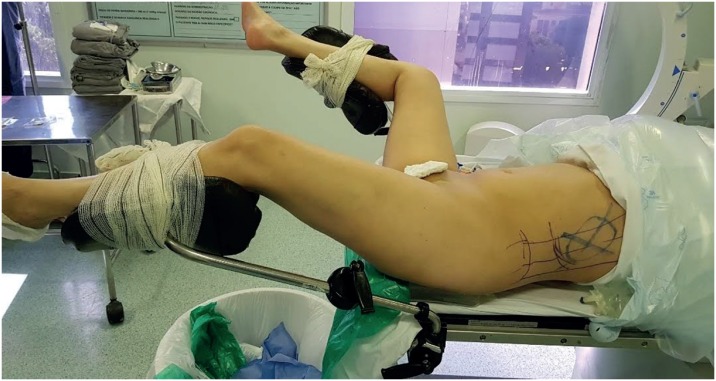
Galdakao position.

PCNL in COMPSUP (Figure-3) followed the procedure described by Falahatkar et al. (12) and modified by Vicentini et al. (13). The patient was placed supine at the edge of the table, with their legs apart and extended and the ipsilateral arm crossed over the thorax. The ureteral catheter was inserted through a rigid cystoscope and the bladder was left empty, with no Foley catheter. GALD (Figure-4) was described by Ibarluzea et al. (14). The patient was placed in an intermediate supine - lateral position with a saline bag placed to raise the flank. The ipsilateral leg was extended and the contralateral leg was abducted and flexed, achieving a modified lithotomy position. The ureteral catheter was placed through a rigid cystoscope. The patients legs were placed in stirrups throughout the procedure.

After positioning and inserting the ureteral catheter, all cases followed the same procedure. A retrograde pyelogram was performed. Under fluoroscopic guidance, the desired calyx was punctured and a guidewire was inserted. The tract was sequentially dilated to 30 Fr using Amplatz dilators® (Boston Scientific, Boston, USA), the Amplatz sheath was positioned and a rigid nephroscope was inserted into the kidney.

Lithotripsy was performed using the LithoClast Master® ultrasonic lithotripter (EMS Electro Medical Systems, Swiss). A flexible nephroscope (Karl Storz, Tuttlingen, Germany) was used at the end of the surgery to investigate RF. Smaller stones were removed intact by retrieval baskets while larger stones were fragmented by Holmium laser. In general, a nephrostomy tube was left after surgery in the following situations: presence of RF, significant intraoperative bleeding, significant collecting system injury, potential persistent bacteriuria or pyonephrosis, and a solitary kidney. In all tubeless PCNL, a ureteral stent was left for 24 to 48 hours after surgery or a Double - J stent was left longer if indicated (e.g., many RF, single kidney, and severe pelvic perforation). Blood transfusions were performed in cases of refractory hypovolemia.

### Statistical analysis

Statistical analysis was performed using SPSS version 23 (SPSS, Inc. Chicago, IL, USA). The results were expressed as the mean ± standard deviation and range. The four PCNL positions were compared using one-way analysis of variance (ANOVA) for continuous variables and the Chi - square test was used for categorical variables. Post hoc analysis after ANOVA used Tukey's procedure. Significance was set at p < 0.05.

## RESULTS

We analyzed 393 PCNL with complete data: 100 patients underwent PCNL in COMPSUP, 94 in VALD, 100 in GALD, and 99 in PRON. Table-1 shows the pre - operative characteristics of those patients. The groups were homogenous.

**Table 1:** Baseline characteristics of patients undergoing PCNL.

		COMPSUP	VALD	GALD	PRON	p value
n (patients)		100	94	100	99	
Age (years), mean ± SD		48.86 ± 12.59	47.86 ± 12.43	50.71 ± 12.12	47.66 ± 12.58	0.293
**Gender**						**0.256**
	Male, %	37.0%	29.8%	43.0%	33.3%	
	Female, %	63.0%	70.2%	57.0%	66.7%	
BMI (kg/m²), mean ± SD		27.40 ± 4.75	27.96 ± 5.79	26.97 ± 4.61	27.10 ± 5.40	0.550
**ASA score**						**0.321**
	ASA 1, %	34.0%	23.4%	26.0%	29.3%	
	ASA 2, %	53.0%	69.1%	57.0%	58.6%	
	ASA 3, %	13.0%	7.4%	16.0%	12.1%	
	ASA 4, %	0%	0%	1.0%	0%	
**Side of the surgery**						**0.201**
	Right, %	55.0%	61.7%	46.5%	52.5%	
	Left, %	45.0%	38.3%	53.5%	47.5%	
Stone largest diameter (mm), mean ± SD		31.06 ± 18.25	29.49 ± 13.61	28.73 ± 11.83	30.34 ± 11.65	0.673
**Guy's Stone Score**						**0.283**
GSS 1, %		15.0%	16.0%	10.0%	22.2%	
GSS 2, %		33.0%	21.3%	27.0%	21.2%	
GSS 3, %		31.0%	42.6%	43.0%	37.4%	
GSS 4, %		21.0%	20.2%	20.0%	19.2%	

Table-2 shows the peri - operative outcomes from PCNL in the different positions.

**Table 2:** Peri-operative results in different positions.

		COMPSUP	VALD	GALD	PRON	TOTAL	p value
Number of PCNL, n		100	94	100	99	393	
Number of puncture tracts, median(range)		1 (1-5)	1 (1-4)	1 (1-3)	1 (1-3)	1 (1-5)	0.381
Operative time (min), mean (±SD)		90.50 (44.36)	111.02 (40.87)	120.85 (49.49)	123.48 (45.14)	111.44 (46.84)	< 0.001
Fluoroscopy time (min), mean (±SD)		12.12 (7.50)	15.60 (8.07)	14.23 (9.47)	15.08 (8.08)	14.23 (8.39)	0.020
Success rate (RF ≤ 4mm), %		58.0%	48.9%	49.0%	47.5%	50.9%	0.428
Stone-free rate, %		43.0%	34.0%	35.0%	37.4%	37.4%	0.565
Blood transfusion rate, %		8.0%	2.1%	3.0%	8.1%	5.3%	0.118
Drop in hemoglobin level (g/dL), mean (SD)		2.22 (1.46)	1.91 (1.28)	1.97 (1.22)	2.34 (1.39)	2.11 (1.35)	0.092
**Complications, n**							**0.001**
	Clavien I	3	1	0	8	12	
	Clavien II	7	5	1	6	19	
	Clavien III	6	5	7	6	24	
	Clavien IV	4	0	1	3	8	
	Clavien V	0	0	0	0	0	
**Total, %**		**20.0%**	**11.7%**	**9.0%**	**23.2%**	**16.0%**	**0.019**
Major complications (Clavien higher or equals to III), %		10.0%	5.3%	8.0%	9.1%	8.1%	0.663
Hospital stay (hours), mean (SD)		53.12 (49.38)	53.66 (36.68)	54.30 (32.99)	67.92 (58.75)	57.23 (45.90)	0.070
Tubeless rate, %		31.3%	21.3%	14.0%	10.1%	19.1%	0.001

We defined success as the absence of RF > 4 mm on POD1 CT and the overall success rate was 50.9%. We were unable to demonstrate a difference in success rates among groups (p = 0.428).

When we compared success rate in each position according to GSS, we observed that the success rate was similar among positions independently of the GSS, but GSS was able to predict the success of PCNL regardless of position (Table-3).

**Table 3:** Success rate (RF ≤ 4mm) in each position according to Guy's Stone Score (GSS).

	GSS 1	GSS 2	GSS 3	GSS 4	TOTAL	p value
**DDH**	93.3%	72.7%	51.6%	19.0%	58.0%	< 0.001
**VALD**	100.0%	50.0%	42.5%	21.1%	48.9%	< 0.001
**GALD**	80.0%	51.9%	55.8%	15.0%	49.0%	0.003
**VENT**	90.9%	42.9%	37.8%	21.1%	47.5%	< 0.001
**Total**	**91.9%**	**56.4%**	**47.0%**	**19.0%**	**50.9%**	**< 0.001**
**p value**	0.347	0.127	0.365	0.958	0.428	

The number of puncture tracts (p = 0.381), blood transfusion rate (p = 0.118), mean drop in hemoglobin level (p = 0.092), and length of hospital stay (p = 0.070) were also similar among groups.

Mean overall operative time was 111.44 minutes. The mean operative time in COMPSUP was = 90.50 minutes, VALD = 111.02 minutes, GALD = 120.85 minutes, and PRON = 123.48 minutes (p < 0.001). When we compared each variable separately, COMPSUP had a significantly shorter operative time than VALD (p = 0.009), GALD (p < 0.001), and PRON (p < 0.001). There was no difference in operative time among VALD, GALD, and PRON.

The mean overall fluoroscopy time was 14.23 minutes. Fluoroscopy times in COMPSUP was = 12.12 minutes, VALD = 15.60 minutes, GALD = 14.23 minutes, PRON = 15.08 (p = 0.020). Again, when we compared positions COMPSUP had a lower fluoroscopy time than VALD (p = 0.020), but it was similar to GALD (p = 0.093) and PRON (p = 0.08). VALD, GALD, and PRON fluoroscopy times were similar.

The tubeless rate also differed among groups (p = 0.001). When we analyzed the groups separately, COMPSUP was associated with higher tubeless rates (p = 0.008) than the other three positions, whereas PRON was negatively associated with lower tubeless rates (p < 0.001).

The overall complication rate was 16.0%, and there were differences among groups (p = 0.019). Complication rates in COMPSUP = 20.0%, VALD = 11.7%, GALD = 9.0%, and PRON = 23.2%. PRON was associated with more complications than the other groups (p = 0.024), and GALD had significantly fewer complications compared to the other three groups (p = 0.026). However, analysis of only major complications (Clavien ≥ 3) revealed no differences among groups.

Table-4 describes the types of complication according to PCNL position.

**Table 4:** Complications according to PCNL position. *

Type of complication	Position				Total
	COMPSUP	VALD	GALD	PRON	
Urinary tract infection	5 (5.0%)	2 (2.1%)	0	2 (1.0%)	9 (2.3%)
Blood transfusion	8 (8.0%)	2 (2.1%)	3 (3.0%)	8 (8.1%)	21 (5.3%)
Tract leakage > 24 h	2 (2.0%)	0	1 (1.0%)	1 (1.0%)	4 (1.0%)
Renal injury	0	1 (1.1%)	0	1 (1.0%)	2 (0.5%)
Pain	3 (3.0%)	1 (1.1%)	0	3 (3.0%)	7 (1.8%)
Calculi migrating to ureter	1 (1.0%)	3 (3.2%)	2 (2.0%)	1 (1.0%)	7 (1.8%)
Colon injury	0	1 (1.1%)	1 (1.0%)	2 (2.0%)	4 (1.0%)
Pleura injury	1 (1.0%)	0	3 (3.0%)	6 (6.1%)	10 (2.6%)
Pseudoaneurysm	2 (2.0%)	0	0	0	2 (0.5%)
Bronchospasm	0	1 (1.1%)	0	0	1 (0.3%)
Pulmonary embolism	1 (1.0%)	0	0	1 (1.0%)	2 (0.5%)
Duodenal injury	0	0	0	1 (1.0%)	1 (0.3%)

* Multiple events may have occurred in a single patient.

## DISCUSSION

The ideal positioning for PCNL remains a matter of controversy. Beyond the debate on prone versus supine positions, variations of supine decubitus make this debate even more complex. Therefore, studies regarding the impact of patient positioning on PCNL outcomes are necessary.

To the best of our knowledge, this is the first study to compare the impact of PCNL positioning performed in three different supine positions and a prone position, on outcomes.

Recently, two meta - analyses showed similar results when comparing prone and supine positions. Yuan et al. (15) reported that PCNL in the prone position was associated with a higher rate of stone clearance than in the supine position. However, the supine position had a shorter operative time and fewer blood transfusions. The length of hospital stays and complication rates were similar. More recently, Falahatkar et al. (16) compared prone and supine positions and showed similar stone - free rate, operation time, hospital stay, complication rate, and urinary leakage. The supine position required less blood transfusion and lower fever rates, suggesting a better safety profile for the supine position. No variations of the supine decubitus were analyzed in both studies.

Positioning in our center was based on surgeon preference. We perform around 300 PCNLs each year in our institution, therefore all surgeons possess a large experience in PCNL. In the beginning of our series, a comparable number of PCNLs were performed in the supine and prone positions. But, as time has progressed, supine procedures have greatly overtaken prone procedures, mainly complete supine position, which is the most used position in our center. Despite similar results between prone and supine position, we observed that surgeons generally prefer the supine position once they begin using that position. This trend was also observed by Sofer et al. (17). Some surgeons preferred to use GALD based on the study of Scoffone et al. (18) which suggested that GALD was the most beneficial position. Moreover, VALD was used mainly because it was the first supine position described and some surgeons learned how to perform supine PCNL using that position, although, as time passes, most of them migrate from GALD and VALD to COMPSUP in our center.

In our study, COMPSUP had the lowest operative and fluoroscopy time. This may be because our staff has more experience performing surgeries in that position. A higher load of cases in COMPSUP may have contributed to quicker PCNL in that position.

Another factor that may have contributed to a faster PCNL in COMPSUP was the absence of saline bags under the flank or leg, such as in VALD and GALD. The bags may prevent quicker renal punctures, increasing fluoroscopy and total operative time. Moreover, the use of saline bags under the flank may make manipulation of the rigid nephroscope more difficult. These two points were the reason for the technical modifications described by Vicentini et al. (13). During PCNL in VALD and GALD, patients stay in a slightly oblique position (15 – 30°) allowing an overlap between the kidney and the spinal column. During COMPSUP, that overlap is avoided. Furthermore, the kidney appears to be more movable in VALD and GALD compared to COMPSUP, which could also contribute to the difference in operative time. When PRON is used, patients need to be positioned and draped twice to perform the PCNL, which could also justify a longer operative time.

Another finding of our study was a higher tubeless rate in COMPSUP and a lower rate in PRON. There is no data in the current literature comparing tubeless rates among PCNL positions. COMPSUP may have had a higher tubeless rate due to faster procedures with fewer adverse events, which encouraged the surgeon to perform a tubeless PCNL.

Despite differences in the overall complication rates, major complications were similar among groups. Marchini et al. (19) used pre - operative CT to show that the kidneys are situated deeper in the abdomen when patients are in a supine position, minimizing the risk of visceral injuries and major complications. We did not observe such a difference in our complication rates. Yuan et al. (15) described an 18.1% complication rate in the supine position versus 20.5% in the prone position. They did not distinguish between different supine positions in that study. Our analysis had an overall complication rate of 16.0%, which was similar to the literature. Variations of the supine position had different complication rates, but similar rates of major complications (Clavien ≥ 3).

The success and stone - free rates in our study were relatively lower than those described in the literature. However, we adopted rigorous criteria for the evaluation of success, including CT on the first POD. We defined success as RF or ≤ 4 mm on POD1 based on the study by Raman et al. (7). We had a large proportion of complex cases, which may have contributed to lower success rates. The CROES PCNL Global Study (20) showed a final success rate of 75.7%. However, in the CROES study the success rates were commonly determined by conventional radiography and after auxiliary procedures, with only 14% of stone - free patients confirmed by CT. Plain radiography has low sensitivity and many RF are missed. However, one of the strengths of our study is that we performed CT on POD1 in 100% of the patients which more precisely reflects the real results of PCNL and allows us to effectively compare what occurred after PCNL in each position, which was the aim of this study.

GSS was created in 2011 by Thomas et al. (21) and confirmed to be a very useful tool for predicting the outcomes of PCNL (22). In our study, success rates were linked to GSS independently of patient positioning.

Our study is not without limitations. Lack of randomization is the main concern. However, prospectively collected data, groups with similar demographics and stone aspects, categorization of patients based on the GSS, regular use of pre - and post - operative CT, and surgeons with extensive experience trained in many different positions make our study relevant. Certainly, a multicentre, randomized study in institutions that regularly use all of the positions, and have a standardized protocol of perioperative care would be ideal. However, until this ideal protocol exists, our study adds more evidence to the literature regarding the impact of different positioning during PCNL for complex kidney stones.

## CONCLUSIONS

Patient positioning during PCNL does not seem to impact the rates of success. Overall complication rate was higher for COMPSUP and PRON, though severe complication rate was similar between groups. Fluoroscopy time was longer for VALD than COMPSUP and similar to other positions. COMPSUP was associated with a lower operative time during the procedure compared with all other positions.

## ABBREVIATIONS

ANOVA: = one - way analysis of variance
ASA score: = American Society of Anesthesiologists physical status classification
BMI: = body mass index
COMPSUP: = complete supine position
CT: = computed tomography
GALD: = Galdakao - modified Valdivia position
GSS: = Guy's Stone Score
PCNL: = percutaneous nephrolithotomy
POD: = post - operative day
PRON: = prone position
RF: = residual fragments
SD: = standard deviation
VALD: = original Valdivia position

## References

[1] Fernström I, Johansson B (1976). Percutaneous pyelolithotomy. A new extraction technique. Scand J Urol Nephrol..

[2] Türk C, Petřík A, Sarica K, Seitz C, Skolarikos A, Straub M (2016). EAU Guidelines on Interventional Treatment for Urolithiasis. Eur Urol..

[3] Goodwin WE, Casey WC, Woolf W (1955). Percutaneous trocar (needle) nephrostomy in hydronephrosis. J Am Med Assoc..

[4] Valdivia Uría JG, Lachares Santamaría E, Villarroya Rodríguez S, Taberner Llop J, Abril Baquero G, Aranda Lassa JM (1987). [Percutaneous nephrolithectomy: simplified technic (preliminary report)]. Arch Esp Urol..

[5] Kumar P, Bach C, Kachrilas S, Papatsoris AG, Buchholz N, Masood J (2012). Supine percuta-neous nephrolithotomy (PCNL): ‘in vogue’ but in which position?. BJU Int..

[6] Vicentini FC, Marchini GS, Mazzucchi E, Claro JF, Srougi M (2014). Utility of the Guy's stone score based on computed tomographic scan findings for predicting percutaneous nephrolithotomy outcomes. Urology..

[7] Raman JD, Bagrodia A, Bensalah K, Pearle MS, Lotan Y (2010). Residual fragments after percutaneous nephrolithotomy: cost comparison of immediate second look flexible nephroscopy versus expectant management. J Urol..

[8] Clavien PA, Sanabria JR, Strasberg SM (1992). Proposed classification of complications of surgery with examples of utility in cholecystectomy. Surgery..

[9] Dindo D, Demartines N, Clavien PA (2004). Classification of surgical complications: a new proposal with evaluation in a cohort of 6336 patients and results of a survey. Ann Surg..

[10] Clayman RV, Surya V, Miller RP, Castaneda-Zuniga WR, Smith AD, Hunter DH (1984). Percutaneous nephrolithotomy: extraction of renal and ureteral calculi from 100 pati-ents. J Urol..

[11] Valdivia Uría JG, Valle Gerhold J, López López JA, Villarroya Rodriguez S, Ambroj Navarro C, Ramirez Fabián M (1998). Technique and complications of percutaneous nephroscopy: experience with 557 patients in the supine position. J Urol..

[12] Falahatkar S, Moghaddam AA, Salehi M, Nikpour S, Esmaili F, Khaki N (2008). Complete su-pine percutaneous nephrolithotripsy comparison with the prone standard technique. J Endourol..

[13] Vicentini FC, Torricelli FC, Mazzucchi E, Hisano M, Murta CB, Danilovic A (2013). Modified complete supine percutaneous nephrolithotomy: solving some problems. J Endourol..

[14] Ibarluzea G, Scoffone CM, Cracco CM, Poggio M, Porpiglia F, Terrone C (2007). Supine Valdivia and modified lithotomy position for simultaneous anterograde and retrograde endourological access. BJU Int..

[15] Yuan D, Liu Y, Rao H, Cheng T, Sun Z, Wang Y (2016). Supine Versus Prone Position in Percutaneous Nephrolithotomy for Kidney Calculi: A Meta-Analysis. J Endourol..

[16] Falahatkar S, Mokhtari G, Teimoori M (2016). An Update on Supine Versus Prone Percutaneous Nephrolithotomy: A Meta-analysis. Urol J..

[17] Sofer M, Tavdi E, Levi O, Mintz I, Bar-Yosef Y, Sidi A (2017). Implementation of supine percutaneous nephrolithotomy: a novel position for an old operation. Cent European J Urol..

[18] Scoffone CM, Cracco CM, Cossu M, Grande S, Poggio M, Scarpa RM (2008). Endoscopic combined intrarenal surgery in Galdakao-modified supine Valdivia position: a new standard for percutaneous nephrolithotomy?. Eur Urol..

[19] Marchini GS, Berto FC, Vicentini FC, Shan CJ, Srougi M, Mazzucchi E (2015). Preoperative planning with noncontrast computed tomography in the prone and supine position for percutaneous nephrolithotomy: a practical overview. J Endourol..

[20] de la Rosette J, Assimos D, Desai M, Gutierrez J, Lingeman J, Scarpa R (2011). The Cli-nical Research Office of the Endourological Society Percutaneous Nephrolithotomy Global Study: indications, complications, and outcomes in 5803 patients. J Endourol..

[21] Thomas K, Smith NC, Hegarty N, Glass JM (2011). The Guy's stone score--grading the com-plexity of percutaneous nephrolithotomy procedures. Urology..

[22] de Souza Melo PA, Vicentini FC, Beraldi AA, Hisano M, Murta CB, de Almeida Claro JF (2018). Outcomes of more than 1 000 percutaneous nephrolithotomies and validation of Guy's stone score. BJU Int..

